# Effect of web‐based education intervention on blood glucose control, self‐care and quality of life in patients with type 2 diabetes: A single‐blinded randomized controlled trial

**DOI:** 10.1111/ijn.13298

**Published:** 2024-08-18

**Authors:** Nurten Terkes, Hicran Bektas, Mustafa Kemal Balci

**Affiliations:** ^1^ Bucak Health School, Department of Internal Medicine Nursing Burdur Mehmet Akif Ersoy University Burdur Turkey; ^2^ Faculty of Nursing, Department of Internal Medicine Nursing Akdeniz University Antalya Turkey; ^3^ Department of Endocrinology and Metabolism Disease Akdeniz University Hospital Antalya Turkey

**Keywords:** nursing, quality of life, self‐efficacy, type 2 diabetes, web‐based education

## Abstract

**Aim:**

This study aimed to assess the effects of web‐based education on blood glucose control, self‐care and quality of life in patients with type 2 diabetes.

**Methods:**

A single‐blinded randomized controlled trial was conducted in accordance with the Consolidated Standards of Reporting Trials (CONSORT) checklist at a university hospital in Turkey. The study included 89 patients with type 2 diabetes who were randomly divided into an intervention group (44) and a control group (45). Participants in the intervention group participated in a 3‐month web‐based education programme.

**Results:**

The findings indicated that there were no significant differences in sociodemographic characteristics and illness features between the intervention and control groups, and both were homogeneous. A statistically significant decrease of 0.71 was observed in the HbA1c (%) level of the intervention group following web‐based education. Following web‐based education, there was a significant difference in body mass index (kg/m^2^) and waist circumferences (cm) between the intervention and control groups. The intervention group displayed significantly improved self‐care and quality of life over the 3‐month period (*p* < 0.05).

**Conclusion:**

This study suggests that web‐based education can enhance the self‐care and quality of life of patients with type 2 diabetes.

## Summary statement

What is already known about this topic?

- Patients with type 2 diabetes frequently struggle to manage their blood glucose levels regularly.
- Continuing regular education of these patients is necessary.
- The use of wireless health applications, including Wi‐Fi, Bluetooth and cellular/mobile phones, in blood glucose monitoring has been shown to have long‐term positive effects in reducing type 2 diabetes complications.

What this paper adds?

- The study demonstrates that nurse‐led interventions through a web‐based education programme can have positive effects on blood glucose levels, self‐care activities, self‐efficacy and quality of life in patients with type 2 diabetes.

The implications of this paper:

- Placing web‐based education within the health system and conducting follow‐ups in collaboration with a multidisciplinary team are recommended.

## 1 INTRODUCTION

Diabetes mellitus (DM) arises from insulin resistance and reduced insulin production and usage and has been linked to unhealthy lifestyle choices and obesity. Type 2 diabetes accounts for approximately 90% of all types of diabetes (Chaney & Clarke, 2014). It is identified by the malfunction of the body in the regulation and utilization of glucose as a source of energy. This chronic condition results in an excessive quantity of sugar circulating in the bloodstream. High blood glucose levels can lead to circulatory, nervous and immune system issues (Mayo Clinic, 2022). The prevalence of type 2 diabetes in the adult population worldwide reached 9.3% in 2019, with approximately 463 million people having diabetes and approximately 4.2 million individuals dying from diabetes and its complications (International Diabetes Federation [IDF], 2020). Turkey has an estimated six million patients with type 2 diabetes. Research from the Turkish DM Epidemiology suggests that the prevalence of type 2 diabetes is 13.7% and almost 27% for patients with impaired glucose tolerance or pre‐DM, respectively (Satman et al., 2013).

If left uncontrolled, type 2 diabetes can cause hyperglycaemia and lead to severe, long‐term complications in various parts of the body, such as the cardiovascular system, eyes, kidneys and nervous system (IDF, 2020). However, if individuals manage type 2 diabetes in its early stages, they can live for many years without chronic complications. However, managing type 2 diabetes is challenging, and healthcare costs increase rapidly once chronic complications arise (Weinstock et al., 2011). For patients with type 2 diabetes, preventing or delaying complications and achieving a healthy and productive life require managing their condition through diabetes education (Beck et al., 2017). The monitoring and education of individuals diagnosed with type 2 diabetes are typically performed on an outpatient basis. However, many individuals with type 2 diabetes experience difficulties in attending regular follow‐up appointments. This challenge is partly due to the limited number of available diabetes nurses and the overcrowded conditions in polyclinics. Healthcare professionals are unable to effectively monitor individuals who cannot attend follow‐up appointments and provide the necessary education (Pazar et al., 2015).

With recent advances in technology, distance learning programmes have been introduced in various healthcare settings and for the education of individuals diagnosed with type 2 diabetes. Recent studies have suggested that technologies such as the internet, computers and mobile phones, which enable remote monitoring and follow‐up at any time, can be effectively employed in the education, care and treatment of patients with type 2 diabetes (Cotter et al., 2014; Murray et al., 2017; Nelson et al., 2016; Ryan et al., 2013). In certain trials, wireless healthcare applications for monitoring blood glucose levels have been documented to generate favourable outcomes and mitigate long‐term complications associated with type 2 diabetes (Sezgin & Cınar, 2013; Weinstock et al., 2011). Self‐efficacy is regarded as a predisposing factor that may deteriorate in chronic conditions such as type 2 diabetes. The elevation of self‐efficacy levels among type 2 diabetes patients can pave the way for blood glucose control (Dehghan et al., 2017). Owing to the COVID‐19 pandemic, the significance of remote patient education and telenursing applications has escalated (Diğin & Kizilcik, 2021). In Turkey, there is currently no evidence‐based web‐based education programme developed in collaboration with a multidisciplinary health team that can be expediently employed in patient education for those afflicted with type 2 diabetes. Therefore, this study aimed to create a web‐based education programme and assess its effects on blood glucose regulation, self‐care, self‐efficacy and quality of life in patients with type 2 diabetes.

### 1.1 Research hypotheses

Compared with the control group, patients with type 2 diabetes in the web‐based education group will have better levels of metabolic variables.

Compared with the control group, patients with type 2 diabetes in the web‐based education group will have higher levels of self‐care and self‐efficacy.

Compared with the control group, patients with type 2 diabetes in the web‐based education group will have higher levels of quality of life.

## 2 METHODS

### 2.1 Study design

The study was a monocentric, single‐blinded, randomized controlled experimental trial.

### 2.2 Design

This study was conducted from May 2017 to April 2018 at the Endocrinology and Metabolic Diseases Polyclinic of a university hospital. A randomized controlled trial was conducted based on the Consolidated Standards of Reporting Trials (CONSORT) 2010 guidelines (Boutron et al., 2008). Participants were randomly assigned to either the intervention or control group.

### 2.3 Sample characteristics

The study included individuals between the ages of 18–65 years who had experienced type 2 diabetes for a minimum of 6 months, utilized insulin, possessed literacy, lacked communication disabilities and did not have any cognitive, physical or mental impairments that may hinder their ability to respond to inquiries. Moreover, participants had computers or mobile devices at home, while those with access to phones and internet facilities were solicited.

**FIGURE 1:**
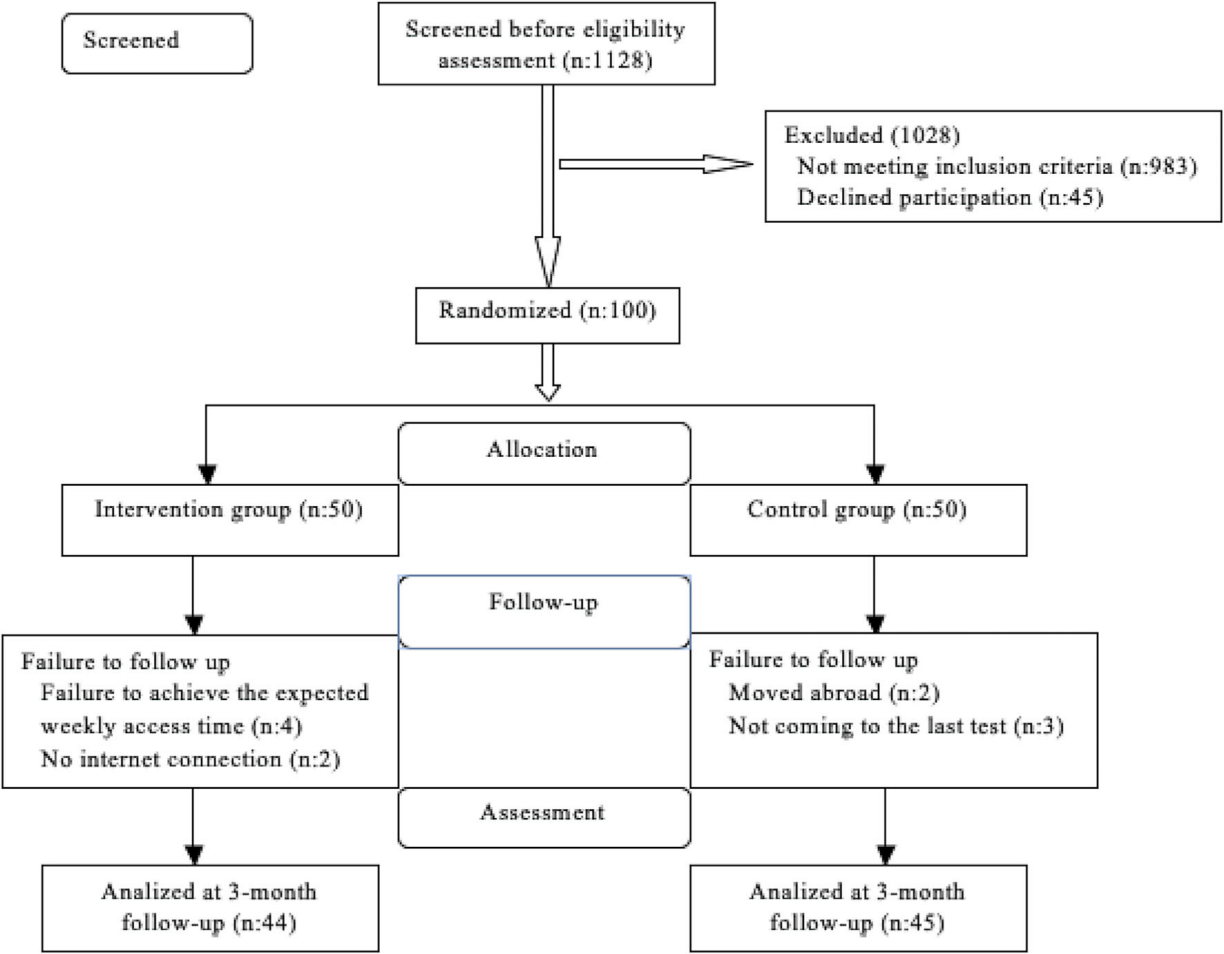
Flow diagram of the randomized controlled study (CONSORT 2010).

A PS software calculation determined that 44 or 68 participants were required, with 80% statistical power and a significance level of 0.05 (Tavsanlı et al., 2013). To allow for potential drops, 50 patients were selected for both the intervention and control groups. During the study, only a portion of the intervention group attained the anticipated weekly website access, and two individuals lost their internet connection. Two individuals from the control group withdrew, and three did not participate in the post‐test. Our study included 89 patients, 44 in the intervention group and 45 in the control group. A graphical representation of this study is shown in Figure 1.

### 2.4 Randomization

Randomization was performed using a web service (https://www.randomizer.org/). The intervention and control groups were selected using a simple randomization method. Participants were unaware of the sections in which they were allocated. Participants were blinded to the study's hypotheses and objectives.

### 2.5 Intervention and control groups

#### 2.5.1 Intervention group

The participants in the intervention group were provided with unlimited and free access to the website. To access the website, they were given a unique login credentials. The structure, content and features of the website were explained to the intervention group. Additionally, the website featured an online forum in which participants could seek answers to their queries. Moreover, the website provides notifications for emergencies related to their concerns. The intervention group participants underwent a 3‐month monitoring period. Additionally, the website was utilized, and reminder messages were sent twice per week via email to support their adherence to prescribed practices and guidelines, ultimately leading to lifestyle changes. Routine maintenance with the web‐based application continued throughout the intervention period. Participants were not required to attend the hospital specifically for data collection, unless they failed to attend their set appointments. The objective was to minimize any extra strain on patients while also enhancing their engagement with the intervention programme.

#### 2.5.2 Control group

The control group did not receive any education and continued with routine follow‐up. After the research was concluded, the website's address was transmitted to the control group via SMS or e‐mails. Subsequently, the developed website was made accessible to the entire country without a password.

### 2.6 Data collection tools

#### 2.6.1 Metabolic control variable form

The Metabolic Control Survey was devised by researchers based on the literature (Heinrich et al., 2012; Moattari et al., 2013; Sezgin & Cınar, 2013; Tutino et al., 2017; Yu et al., 2014). It comprises a nine‐item questionnaire that gathers clinical data, including height (cm), weight (kg), body mass index (kg/m^2^) and blood pressure (mmHg), as well as laboratory results such as blood glucose (mg/dL), HbA1c (%), cholesterol (mg/dL) and triglyceride (mg/dL) levels.

#### 2.6.2 The Diabetes Self‐Care Activities Questionnaire

Toobert et al. (2000) designed this questionnaire to evaluate the self‐care practices of individuals with diabetes. Kav et al. (2010) validated and evaluated the reliability of the same questionnaire in Turkey. The study revealed that the total Cronbach's alpha value of the Turkish version was 0.72. The scale includes 11 items, and the overall scores varied from 0 to 7. Increased scores indicate an elevated level of self‐care activity (Kav et al., 2010).

#### 2.6.3 The Self‐Efficacy Scale

This scale was created by Bijl et al. (1999), and its Turkish validity and reliability were tested by Kara et al. (2006). The total Cronbach's alpha for the Turkish version was 0.88. The scale comprises 20 items rated on a 5‐point Likert scale. Responses are assessed on the basis of a 1‐point score for ‘No, I'm not sure’ and a 5‐point score for ‘Yes, I'm sure’. The total scale score ranges from 20 to 100. High total scores on the scale suggest good self‐efficacy in managing diabetes (Kara et al., 2006).

#### 2.6.4 The Diabetes Quality of Life Scale

Jacobson et al. (1994) developed this scale to assess the quality of life of people with diabetes. Yildirim et al. (2007) conducted the validity and reliability study of the scale in Turkey. The Cronbach's alpha for the Turkish version was 0.89. The scores on the scale range from 46 to 230. A high total score indicates good quality of life (Yildirim et al., 2007).

### 2.7 Statistical analysis

The data obtained from the study were analysed using IBM SPSS Statistical Package for Social Science version 23.0. The numeric results were expressed as mean ± SD, and categorical results were expressed as frequencies and percentages. Categorical variables were compared using the chi‐square test. Variables were tested for normal distribution using a one‐sample Kolmogorov–Smirnov test to use a parametric or non‐parametric test. All the variables were normally distributed; therefore, parametric statistical methods were used. Comparisons of the differences between scores at the end of the 3 months and at baseline were performed between the intervention and control groups using an independent samples *t* test for all variables. Accordingly, changes in scores between the two time points regarding self‐care, self‐efficacy, quality of life and metabolic control variables were calculated for each group, and the mean changes were compared between the two groups. An intention‐to‐treat analysis was performed due to the loss of data in the intervention and control groups. Expert opinions were acquired regarding the content of web‐based education, and Kendall's coefficient of concordance was calculated. The Cronbach's alpha coefficient was used to assess the reliability of the scale. A two‐sided probability value of <0.05 was considered statistically significant.

### 2.8 Ethical approval

The Clinical Research Ethical Committee of the Faculty of Medical Sciences (385.AKD.23.12.2015) approved the project in accordance with ethical guidelines. Informed consent was obtained from all participants in the Endocrinology Department. The objective of the study was explained to all participants who were informed of their right to refuse participation or withdraw from the study at any time. Permission to use the scales was obtained through email correspondence with developers.

## 3 RESULTS

Analyses were conducted to compare differences between the intervention and control groups. The distribution of sociodemographic characteristics such as age, gender, education, occupational status, employment status and the presence of someone providing care showed no significant differences between the intervention and control groups (*p* > 0.05). For all variables, the sociodemographic characteristics of the intervention and control groups were similar (see Table 1). This analysis was conducted by a proficient statistician, with the researchers not involved in the data analysis.

**TABLE 1:** Sociodemographic characteristics of patients with type 2 diabetes (*n* = 89).

Sociodemographic characteristics	Intervention group (*n*: 44) *n* (%)	Control group (*n*: 45) *n* (%)	*p*
Age (years) ( $\bar{X}\pm \mathrm{SD}$)	51.93 ± 8.48	52.55 ± 7.49	0.873
Gender			
Female	23 (52.3%)	25 (55.6%)	0.883
Male	21 (47.7%)	20 (44.4%)	
Education			
Literate	15 (34.1%)	22 (48.9%)	0.465
Primary	4 (9.1%)	5 (11.1%)	
Secondary	13 (29.5%)	11 (24.4%)	
High	12 (27.3%)	7 (15.6%)	
Occupational status			
Retired	17 (38.6%)	16 (35.6%)	0.138
Homemaker	13 (29.5%)	19 (42.2%)	
Self‐employed	14 (31.9%)	10 (22.2%)	
Employment status			
Employed	13 (29.6%)	10 (22.2%)	0.493
Unemployed	31 (70.5%)	35 (77.8%)	
Presence of someone who supports their care			
Yes	23 (52.3%)	26 (57.8%)	0.602
No	21 (47.7%)	19 (42.2%)	

### 3.1 Change of metabolic control variables according to time and group

Based on pretest–post‐test analysis of the intervention and control groups at the baseline and at the conclusion of the third month, the mean fasting plasma glucose (FPG, mg/dL), HbA1c (%), body mass index (kg/m^2^), waist circumference (cm) and systolic blood pressure (mmHg) levels in the intervention group showed significant statistical differences (*p* < 0.05). Although there was a statistically significant variance in mean body mass index (kg/m^2^) and waist circumference (cm) (*p* < 0.05), there were no statistically significant differences between the intervention and control groups at baseline and after 3 months in mean FPG (mg/dL), HbA1c (%), systolic blood pressure (mmHg), diastolic blood pressure (mmHg), direct LDL cholesterol (mg/dL) and triglyceride levels (mg/dL) (*p* > 0.05) (Table 2).

**TABLE 2:** Change of metabolic control variables according to time and group (*n* = 89).

Metabolic control variables	Intervention group (*n*: 44) $\bar{X}$±SD		Time *p*	Control group (*n*: 45) $\bar{X}$±SD		Time *p*	Intergroup difference analysis *p*
	Baseline	3rd month		Baseline	3rd month		
Fasting plasma glucose (mg/dL)	179.2 ± 89.8	144.4 ± 51.0	0.01	167.6 ± 68.7	157.9 ± 47.9	0.83	0.073
HbA1c (%)	8.11 ± 1.67	7.60 ± 1.17	0.00	8.39 ± 1.71	8.03 ± 1.40	0.23	0.233
Body mass index (kg/m^2^)	32.9 ± 6.0	32.1 ± 6.0	0.00	31.9 ± 6.1	31.8 ± 6.3	0.56	0.006
Waist circumference (cm)	107.7 ± 13.8	106.5 ± 13.4	0.00	104.0 ± 17.0	106.3 ± 13.3	0.30	0.002
Systolic blood pressure (mmHg)	132.8 ± 17.8	127.4 ± 11.3	0.00	138.2 ± 19.6	136.1 ± 19.7	0.46	0.266
Diastolic blood pressure (mmHg)	80.6 ± 10.5	79.3 ± 8.6	0.77	82.7 ± 13.6	79.8 ± 12.7	0.08	0.375
Direct LDL cholesterol (mg/dL)	106.0 ± 37.7	102.3 ± 40.8	0.21	99.1 ± 37.5	96.4 ± 30.2	0.62	0.640
Triglyceride (mg/dL)	177.0 ± 103.7	165.9 ± 73.3	0.63	177.0 ± 114.9	179.7 ± 139.9	0.98	0.616

### 3.2 Self‐care, self‐efficacy and quality of life changes in mean scale scores according to time and group

There were notable differences within and between the groups regarding the average scores on the Diabetes Self‐Care Activities Questionnaire, Diabetes Self‐Efficacy Scale and Diabetes Quality of Life Scale (*p* < 0.05). Individuals in the intervention group reported an increase in their mean scores, while those in the control group experienced a decrease. Statistically significant differences were observed in the mean scores of the groups on the Diabetes Self‐Care Activities Questionnaire, Diabetes Self‐Efficacy Scale and Diabetes Quality of Life Scale (*p* < 0.05) (Table 3).

**TABLE 3:** Change in the mean scale scores according to the time and group (*n* = 89).

Scales	Intervention group (*n*: 44) $\bar{X}$±SD		Time *p*	Control group (*n*: 45) $\bar{X}$±SD		Time *p*	Intergroup difference analysis *p*
	Baseline	3rd month		Baseline	3rd month		
Diabetes self‐care activities	3.64 ± 0.90	5.51 ± 0.91	<0.001	3.67 ± 1.07	3.47 ± 0.87	0.032	<0.001
Diabetes self‐efficacy	3.36 ± 0.49	4.23 ± 0.48	<0.001	3.51 ± 0.50	3.22 ± 0.54	<0.001	<0.001
Diabetes quality of life	4.02 ± 0.33	4.38 ± 0.24	<0.001	4.22 ± 0.36	4.11 ± 0.35	<0.001	<0.001

## 4 DISCUSSION

Diabetes self‐management education and assistance are a part of lifestyle management, which is a basic component of diabetes care. To improve diabetes care, patients and healthcare providers should collaborate on optimizing lifestyle from the time of the initial comprehensive medical evaluation, through all follow‐up appointments and evaluations, and during the assessment of complications and management of comorbid conditions. Every individual with diabetes should take part in diabetes self‐management support to help them put the skills and behaviours they need for ongoing self‐management into practice and in diabetes self‐management education to help them acquire the knowledge, abilities and skills needed for diabetes self‐care (American Diabetes Association [ADA], 2018). Our study focused on the effect of web‐based education on diabetes management and its complications in individuals with type 2 diabetes.

Electronic education programmes offer communication and counselling services for patients to directly engage with healthcare professionals, thereby boosting their metabolic control (Moattari et al., 2013). In this study, there was no significant difference in mean HbA1c levels between the intervention and control groups; however, there was a statistically significant difference of 0.71 in time‐dependent HbA1c levels between the intervention and control groups at baseline and after 3 months. Sezgin and Cınar's (2013) study revealed that the intervention group receiving web‐based education experienced a decrease in mean HbA1c levels from 8.0% to 6.9%, while the control group exhibited an increase from 8.1% to 8.6%. In another study conducted using the internet, Ryan et al. (2013) discovered a statistically significant difference with a 0.62% decrease in HbA1c levels in the intervention group at the end of 13 months. Widyanata and Arifin (2019) demonstrated a notable correlation between technology‐based education and HbA1c level. Our investigation corroborates these findings. Self‐care management of type 2 diabetes, as a chronic disease, may benefit from additional educational tools in addition to web‐based resources. In addition, monitoring of type 2 diabetes metrics is crucial for long‐term management.

The intervention group demonstrated a significant improvement in both body mass index (kg/m^2^) and waist circumference (cm) following web‐based education at baseline and after 3 months. Obesity increases the risk of type 2 DM. Losing 5% to 10% of body weight via 30 min of daily physical activity leads to a 58% decrease in complications associated with type 2 diabetes (Widyanata & Arifin, 2019). Numerous investigations have indicated that online education regarding diabetes can have advantageous outcomes for individuals with type 2 diabetes in terms of body mass index (BMI) (Avdal et al., 2020; Beck et al., 2017; Hansel et al., 2017).

In our study, a statistically significant decrease was observed in mean systolic blood pressure (mmHg) in the intervention group. In the literature, two studies found no significant difference in terms of diastolic blood pressure (mmHg), while one found a significant difference in terms of systolic blood pressure (Murray et al., 2017; Sezgin & Cınar, 2013). Another study reported a statistically significant difference between the intervention and control groups in blood pressure levels (Morrow et al., 2013). Pal et al. (2013) stated that web‐based interventions could improve the health outcomes of patients with type 2 diabetes.

Individuals with low self‐efficacy exhibit inadequate self‐care behaviours, which leads to poor diabetes management (ADA, 2018). In our study, web‐based education significantly improved self‐care practice in the intervention group. Numerous studies conducted on patients with type 2 diabetes have shown that web‐based education improves self‐care practices and disease knowledge (Pal et al., 2013; Rondags et al., 2016; Yu et al., 2014). According to a systematic review conducted by Cotter et al. (2014), online education has a beneficial impact on self‐care. Similarly, Cassimatis et al. (2014) also discovered favourable outcomes and suggested that a web‐based system designed to support self‐management of emotional and psychological needs holds promise for attaining high acceptability and perceived benefit. The results also indicate that it is crucial to provide diabetes type 2 patients with web‐based education and to maintain regular follow‐up and monitoring to realize its full potential and proper usage.

The web‐based education provided to patients has been shown to have a positive effect on their quality of life. A meta‐analysis revealed that web‐supported education is advantageous for individuals with type 2 diabetes, reducing complications and enhancing the quality of life (Shen et al., 2018). One study established that participants in the intervention group displayed notably higher post‐test scores on the Diabetes Quality of Life Scale than those in the control group (Tavsanlı et al., 2013). Ebert et al. (2017) found that web‐based self‐management provided to the intervention group had statistically significant results on the quality of life of individuals diagnosed with type 2 diabetes.

### 4.1 Limitations

Our study has several limitations. The trial sample was limited to patients with type 2 diabetes, thereby limiting the generalizability of the findings to other types of diabetes. Additionally, the participation of only those with internet access is a potential limitation of this trial. Finally, the potential impact of time should be considered, and a repeated study was conducted to determine the persistence of changes after 6 months or 1 year. Finally, the conclusions of this study were valid only for the participants included in the study.

## 5 CONCLUSION

This study showed that web‐based education had a positive effect on blood glucose control, self‐care, self‐efficacy and quality of life in patients with type 2 diabetes. The fact that type 2 diabetes is a chronic disease increases the need for and importance of education for diabetes management and prevention of complications. Web‐based education is especially valuable during pandemics, disaster situations and inadequate access to healthcare services. In terms of the continuity of the education of patients with type 2 diabetes, it is important to disseminate web‐based education and develop systems that nurses can follow patient education and follow‐up through web‐based applications. Web‐based applications prepared in line with evidence‐based practices should be included in health services and available free of charge to patients. Moreover, web‐based education enables patients to receive ongoing education regardless of location and time while allowing nurses to provide support. This could potentially lead to a decrease in hospitalization rates. Future studies are needed to test the long‐term effects of web‐based education.

## AUTHORSHIP STATEMENT

All authors contributed to the interpretation, writing and approval of the final manuscript.

## CONFLICT OF INTEREST STATEMENT

The authors declared no potential conflicts of interest for the research, authorship and/or publication of this article.

## Data Availability

The data that support the findings of this study are available from the corresponding author upon reasonable request. Data extraction and connection to access data is not possible.
